# Rhabdomyolysis in a Hospitalized 16-Year-Old Boy: A Rarely Reported Underlying Cause

**DOI:** 10.1155/2016/7873813

**Published:** 2016-11-08

**Authors:** Rishika Singh, Dilip R. Patel, Sherry Pejka

**Affiliations:** ^1^Department of Pediatric and Adolescent Medicine, Western Michigan University Homer Stryker MD School of Medicine, Kalamazoo, MI, USA; ^2^Bronson Children's Hospital, Kalamazoo, MI, USA

## Abstract

Rhabdomyolysis can occur because of multiple causes and account for 7% of all cases of acute kidney injury annually in the United States. Identification of specific cause can be difficult in many cases where multiple factors could potentially cause rhabdomyolysis. We present a case of 16-year-old male who had seizures and was given levetiracetam that resulted in rhabdomyolysis. This side effect has been rarely reported previously and like in our case diagnosis may be delayed.

## 1. Case Report

A 16-year-old, previously healthy, well-built male presented to the hospital after having two witnessed generalized tonic clonic seizures. The first seizure occurred at school and lasted for 2 minutes. The second seizure occurred in the emergency department and lasted for 2.5 minutes. The patient was given lorazepam and intravenous levetiracetam. He was conscious and aware of his surroundings between the two seizures and 1 minute after the second seizure. There was no reported fall or other trauma to the patient before or during the seizure. Initial labs revealed serum bicarbonate of 18 mmol/L, normal serum electrolytes, and creatinine. Other negative studies included urinalysis, urine drug screen (screened for opiates, barbiturates, phencyclidine, amphetamines, cocaine, cannabinoids, and benzodiazepines), ethanol, acetaminophen, and salicylate levels in the blood. Patient was given a normal saline bolus followed by maintenance fluid. In consultation with neurology, levetiracetam level was not obtained.

Levetiracetam was continued at 750 mg orally every 12 hours. The following day, patient developed back pain. It was located lateral to lumbosacral spine on both sides and the area was tender to palpation. Creatine kinase (CK) drawn at this time was 565 U/L; creatinine (Cr) was elevated at 2.2 mg/dL. Repeat urinalysis was performed and was positive for myoglobin, negative for protein, red blood cells, white blood cells, and bacteria. At this point, it was thought that rhabdomyolysis was secondary to the seizure and the treatment was continued. We omitted potassium from the fluids and increased the rate to 200 mL/hr to provide more aggressive hydration. We continued to check creatine kinase and renal function every 6 hours. Patient's back pain continued to worsen. The pain was now also involving his upper back and thighs. He was requiring narcotics for pain control. Creatine kinase continued to rise exponentially and was 15,111 U/L on day 4; creatinine remained elevated with only mild fluctuations. We discontinued levetiracetam and switched to devalproex sodium on day 5. This was followed by improvement in patient's back pain the next day.

Patient's back pain completely resolved in 2 days. There was a steady and quick decline in the creatine kinase level and creatinine. Creatinine normalized by day 7 and creatine kinase by day 10. Patient was discharged home on devalproex sodium. He has been doing well since discharge. Creatine kinase levels have been checked multiple times following discharge and have remained normal. Subsequent renal function tests have also remained normal.

## 2. Discussion

In this patient our diagnosis was rhabdomyolysis and subsequent acute kidney injury (AKI) due to levetiracetam. Rhabdomyolysis is a common clinical syndrome that results from the rapid breakdown of skeletal muscle fibers, which leads to leakage of potentially toxic cellular contents into the systemic circulation [1]. It ranges from an asymptomatic illness with elevation of creatine kinase level to a life-threatening condition associated with extreme elevations in creatine kinase, electrolyte imbalances, acute renal failure, and disseminated intravascular coagulation [2]. The classic reported triad of symptoms includes muscle pain, weakness, and dark urine, although more than 50% of the patients do not complain of muscle pain or weakness and the classic triad is seen in <10% patients [3]. Nonspecific symptoms like fever, malaise, nausea, and vomiting may be present [2].

More than 25,000 cases are reported annually in the USA, which account for 7% of all cases of AKI [4]. Acute renal failure from rhabdomyolysis in children is much less common (~5%) than the risk reported for adults [5]. Theoretically, any form of muscle damage can lead to rhabdomyolysis. In the pediatric population, viral myositis, trauma, connective tissue disorders, exercise, and drug overdose are responsible for much of the rhabdomyolysis seen in these patients [2, 5]. There are close to 200 medications and toxins known to cause skeletal muscle injury [4, 6]. In some cases a cause of rhabdomyolysis cannot be clearly established [7].

Diagnosis is based on the elevation of creatine kinase >10 times the normal value. It rises within 2–12 hours of muscle injury, peaks around 24–72 hours, and then declines over the next 3–5 days [1, 8]. Myoglobinuria can be present which may be discovered as positive urine dipstick test for blood in absence of red blood cells. Other electrolyte derangements may be present including hyperkalemia, hyperphosphatemia, hypocalcemia, and lactic acidosis [4]. Renal function should be assessed since there is a risk of AKI [1]. Electrocardiogram should be obtained if electrolyte abnormalities are present. Other complications that can occur in patients with rhabdomyolysis are compartment syndrome and disseminated intravascular coagulation [2].

The main objective in management is to prevent or treat AKI which is achieved mainly by aggressive hydration [2]. Due to accumulation of water in the muscle fibres and subsequent hypovolemia, fluid resuscitation is imperative to prevent prerenal azotemia [4]. Normal saline should be used without potassium or lactate. Intravenous infusion should be given at 1.5 L/hr to maintain urine output of 200 mL/hr and continued until creatine kinase value has declined to <1000 IU/L [3, 9]. If the offending agent is suspected to be a medication, it should be discontinued promptly [4]. Evidence supporting the use of mannitol and/or bicarbonate comes mostly from animal studies and is inconsistent and conflicting. Recommendations for the use of bicarbonate to alkalinize the urine are based on retrospective studies in adults with no supporting data in the literature on pediatric patients [10]. Renal function and electrolytes should be monitored closely. Some patients with severe AKI may need hemodialysis. Prompt fasciotomy is required for patients with compartment syndrome [4].

Levetiracetam is generally considered safe and a commonly used antiseizure medication in pediatric population. Rhabdomyolysis is not a well-documented or listed adverse effect on this drug's profile but rare case reports have implicated levetiracetam as a cause of rhabdomyolysis [11, 12].

Based on this previously reported side effect of levetiracetam and temporal relationship between clinical and lab improvement with discontinuation of the drug, we surmise that rhabdomyolysis in our patient was due to this drug. In patients with elevated creatine kinase, drug induced rhabdomyolysis should always be considered.
